# Genetic disruption of *Capns1* impairs metastatic phenotypes in murine mammary carcinoma cells

**DOI:** 10.17912/micropub.biology.002124

**Published:** 2026-06-09

**Authors:** Danielle Harper, Ivan Shapovalov, Samantha Cockburn, Anya Aaron, Yan Gao, Peter A Greer

**Affiliations:** 1 Pathology and Molecular Medicine, Queen's University

## Abstract

Calpains are a family of 15 calcium-activated cysteine proteases that have emerged as potential antimetastatic targets in breast cancer. Calpain-1 and calpain-2 are ubiquitously expressed heterodimers composed of unique catalytic subunits (encoded by *Capn1* and *Capn2* , respectively) and a common regulatory subunit (encoded by *Capns1* ). Genetic disruption of *Capns1* abolishes calpain-1 and calpain-2 activity. Using CRISPR-Cas9 mediated *Capns1* knockout, we validated calpains-1/2 as promising therapeutic targets in a mouse model of mammary carcinoma. *Capns1* knockout impaired cell invasion by 53 ± 10% *in vitro* and reduced lung metastasis by 68 ± 12% in an orthotopic engraftment mouse model.

**Figure 1. Capns1 knockout impairs AC2M2 invasion in vitro and metastatic progression in vivo:** **A** ) Validation of genetic calpain-1/2 disruption in AC2M2 cells by immunoblotting (upper) and casein zymography (lower). ( **B** ) Relative distance invaded from 3D AC2M2 spheroids at 48 (left) and 72 hours (right). ( **C** ) Representative images of spheroids at 48 hours. ( **D** ) Resect-and-wait orthotopic engraftment metastasis model workflow ( **E** ) Tumour growth curves (F) Relative tumour growth rates *in vivo* . ( **G** ) Lung metastatic burden 10 days post tumour resection, normalised to WT control. ( **H** ) Representative whole mount images of lungs with GFP signal corresponding to metastatic lesions.

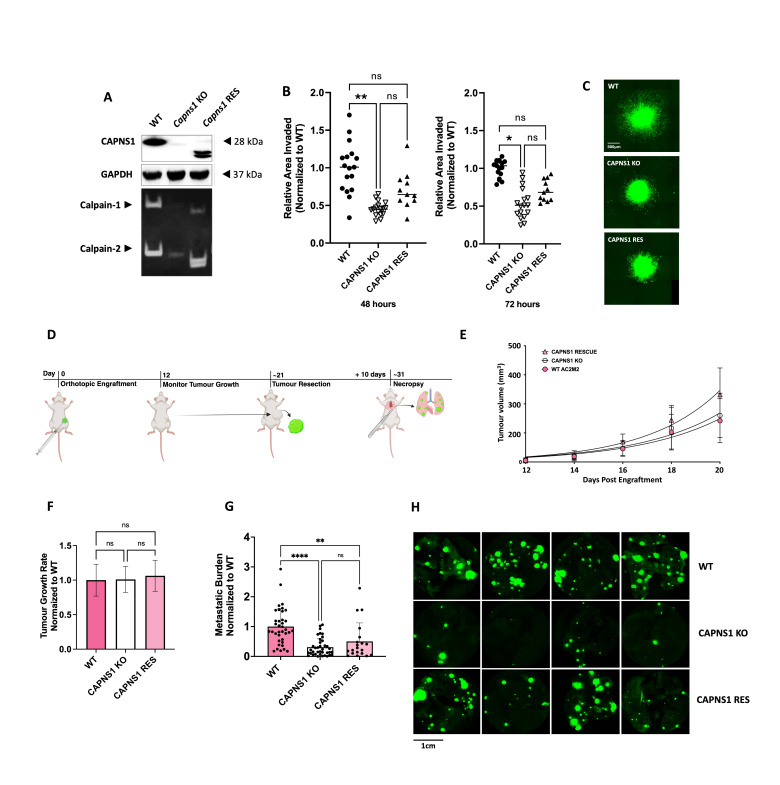



## Description

Triple-negative breast cancer (TNBC) is a heterogenous breast cancer subtype characterized by a lack of estrogen receptor (ER), progesterone receptor (PR), and human epidermal growth factor receptor 2 (HER2) expression. Across all breast cancer subtypes, progression from localized to metastatic disease is associated with 76.8% reduction in 5-year survival (Ellison & Saint-Jacques, 2023). The deadly consequences of metastatic progression are exacerbated for TNBC patients, with 5-year survival for stage IV disease at just 12.1% (Surveillance Research Program, 2025). This underscores an urgent need for therapies which can prevent or slow the metastatic progression of TNBC.

A growing number of preclinical and translational studies have implicated calpain proteases in cancer (Shapovalov et al., 2022; Storr et al., 2011). Human calpains are a family of 15 calcium-activated cysteine proteases (Ono & Sorimachi, 2012). The two most widely studied isoforms, calpain-1 and calpain-2 (referred to as calpain-1/2), are ubiquitously expressed heterodimers consisting of a unique large catalytic subunit, encoded by *CAPN1* or *CAPN2* genes respectively, and an obligate common regulatory subunit, encoded by *CAPNS1* (Wheelock, 1982). Genetic disruption of *CAPNS1* is associated with a loss of calpain-1 and calpain-2 activity (Arthur et al., 2000). At present, there are no clinically approved calpain inhibitors available. While several active-site directed calpain inhibitors have been developed, they produce off-target effects on other proteases and lack specificity for calpains, let alone individual calpain isoforms (Ono et al., 2016). We have employed genetic approaches to validate calpain-1/2 as promising therapeutic targets in TNBC.

We previously reported that genetic disruption of *CAPNS1* impaired human MDA-MB-231 TNBC cell migration *in vitro* and attenuated lung metastasis in an orthotopic xenograft mouse model (Harper et al., 2025). Here, we demonstrate that CRISPR-Cas9 mediated knockout of *Capns1* produces similar effects in an allogeneic mouse model using the AC2M2 murine mammary carcinoma cell line. AC2M2 is a highly metastatic variant of the TNBC-like SP1 mammary adenocarcinoma cell line derived in the laboratory of Dr. Bruce Elliott at Queen’s University (Elliott et al., 2005). Following validation of CRISPR-Cas9 mediated *Capns1* knockout (KO) and gene add-back rescue (RES) by immunoblotting and casein zymography ( Figure 1A ), we employed a spheroid invasion assay to a observe how loss of calpain-1/2 activity affects 3D-invasion. Lung metastasis was evaluated *in vivo* using an orthotopic engraftment, resect-and-wait-mouse model.

To assess the impact of *Capns1* KO on the ability of AC2M2 cells to invade the extracellular matrix, WT, *Capns1* KO, and *Capns1* RES AC2M2 spheroids were established in cell-repellent, round-bottom 96-well plates, and subsequently embedded into a 25% Matrigel matrix on an Ibidi chamber slide. As illustrated in Figure 1 panels B and C, *Capns1* KO was associated with a statistically significant reduction in total area invaded at 48 and 72 hours compared to WT, (48 hours: KO vs WT: 46 + 9%, p=0.0053; 72 hours: KO vs WT 53 + 10%, p=0.0130; one-way ANOVA with Tukey’s multiple-comparisons test). While *Capns1* RES trended towards increased invasiveness compared to *Capns1* KO, there were no statistically significant differences in total area invaded at 48 or 72 hours (p=0.1159 and p=0.3606, respectively). Furthermore, there were no significant differences in total area invaded at 48 or 72 hours between WT and *Capns1* RES (p=0.1008 and p=0.0998, respectively).

In our resect-and-wait model orthotopic engraftment model, tumours were resected by recovery surgery at 500mm ^3^ , rather than at a specific timepoint post-engraftment. This was ensure there were no significant differences in tumour volumes between genotypes at resection. If *CAPNS1* knockout had an effect on tumour growth, we wanted to control for the possibility that a lower lung metastatic burden could be attributed to a smaller tumour size, rather than direct calpain mediated effects on metastatic dissemination. In our AC2M2 model, there were no significant differences in tumour growth rates, as demonstrated in figures 1E and 1F. The tumour growth curve illustrated in Figure 1E represents average tumour volumes between the day of first palpable tumour measurements (day 12) and the first resection surgery on day 21. Tumours were resected at various timepoints between day 21 and day 24. The tumour growth rate for each tumour was calculated using a non-linear regression between day 12 and the day of resection, and genotype averages were compared using a one-way ANOVA (p=0.6362).

As illustrated in Figure 1G, *Capns1* KO was associated with a 68 + 12% reduction in metastatic burden compared to WT, which was statistically significant (p<0.0001, one-way ANOVA with Tukey’s multiple-comparisons test). *Capns1* RES was able to restore metastatic burden to 50 + 14% that of WT, but this was significantly less than WT (WT vs. *Capns1* RES, p=0.0017), suggesting complete rescue was not achieved *(Capns1* KO vs. *Capns1* RES, p=0.4961). Representative whole-mount images of lungs are shown in Figure 1F, where each hyperintense green nodule represents a metastatic lesion.

In summary, *Capns1* KO significantly impaired AC2M2 invasion *in vitro,* and lung metastasis *in vivo.* Despite restoring calpain-1 and calpain-2 proteolytic activity ( Figure 1B ), *Capns1* rescue did not completely restore the WT phenotypes in either experiment. One possible explanation for this observed failure is off-target CRISPR-Cas9 activity, which could be mitigated by testing multiple guide RNAs or utilising alternative methods of genetic disruption including shRNA knockdown. It is also worth noting that the *Capns1* rescue construct lacked 129 nucleotides encoding 43 amino acids comprising most of the N-terminal glycine rich region of CAPNS1. Functional roles for the glycine rich domain of CAPNS1 remain to be fully elucidated, but there is evidence to suggest it plays a role in subcellular localization. The N-terminal glycine rich region of CAPNS1 has been proposed to play a role in phosphatidylinositol (PI) initiated autolysis and subsequent activation of calpain-1 at the plasma membrane (Imajoh et al., 1986). Additionally, anchoring of calpain-2 at the plasma membrane via phosphatidylinositol 4,5-bisphosphate (PIP _2_ ) binding has been reported to promote its activation (Leloup et al., 2010). Therefore, the physiological behaviour of our rescued calpain protein *in vivo* may not be reflected by *in vitro* zymography analysis, where excess CaCl _2_ in cell lysates artificially enhances proteolytic activity. Nevertheless, our findings are consistent with previous reports demonstrating the antimetastatic effects of genetic calpain-1/2 disruption (Grieve et al., 2016; Harper et al., 2025; Jeon et al., 2025; MacLeod et al., 2018), providing rationale for the development of selective calpain-1/2 inhibitors.

There are currently no calpain inhibitors approved for clinical use. While there are a number of inhibitors used in research applications, they lack specificity for individual calpain isoforms and inhibit other proteases, including the proteasome, cathepsins and caspases (Ono et al., 2016). Furthermore, systemic calpain inhibition may produce unwanted side effects. Germline loss of *Capns1* or *Capn2* is embryonic lethal but inducible or tissue specific *Capns1* knockout appears to be well tolerated in adult mice (Arthur et al., 2000; Dutt et al., 2006; Takano et al., 2011; Kumar et al., 2014). Like many cytotoxic cancer therapies, calpain inhibitors would likely be contraindicated for pregnant women, especially during the first trimester. In mice, dogs, and humans, loss of function *CAPN1* mutations result in a predisposition to cerebellar ataxia and muscle wasting (Wang et al. 2016). While calpain-3 is the major isoform expressed in muscle tissue, loss of *Capns1* has also been shown to contribute to muscular dystrophy in aged mice (Piper et al., 2020). These considerations may be useful for informing hypothetical future decisions regarding the duration of calpain-inhibitor treatment as an anti-metastatic approach.

For short-term prevention of metastatic progression, for example between initial diagnosis and surgery, or during neoadjuvant immunotherapy, a calpain inhibitor may be well tolerated. However, if used as a long-term approach to limit further progression in the metastatic setting, the risk of adverse effects may increase. In summary, the ubiquitous nature of calpain expression raises concerns of off-target effects associated with calpain disruption, but current evidence from preclinical models suggests that calpain inhibitors are likely to be well tolerated, at least in the short term. However, in order to address these concerns, we require a selective calpain-1/2 inhibitor with pharmacokinetic properties that permit *in vivo* applications. Calpain proteases remain a promising anti-metastatic therapeutic target, and the development of selective calpain-1/2 inhibitors has the potential to improve clinical outcomes for TNBC patients.

## Methods

*Genetic manipulation of Capns1*

CRISPR-Cas9 mediated knockout (KO) of mouse *Capns1* in AC2M2 cells was achieved by transduction with lentivirus produced in HEK293T cells from psPAX2 and pMD.2G, and lentiCRISPRv2-Neo plasmids as previously described (Sanjana et al., 2014). The gene targeting guide RNA (m- *Capns1* gRNA) sequence (5’-gtgctcggaggcctgatcag-3’) was cloned into the BsmBI restriction site of lentiCRISPRv2-Neo lentiviral shuttle vector (Primers, Forward: 5’-caccgtgctcggaggcctgatcag-3’, Reverse: 5’-aaacctgatcaggcctccgagcac -3’). Neomycin-selected transduced cells were diluted in 96 well plates to produce monoclonal populations of *Capns1* KO AC2M2s. Using AC2M2 cDNA as a template, *Capns1* was amplified by PCR and cloned into the *PmeI* restriction site of the pMSCV-puro retroviral vector (Primers, Forward: FW: 5’-cgacgtttaaactcatgttcttggtgaattcgttcttg-3’, Reverse: 5’- gtccgtttaaacactcagggtgcagcaaggtgtggcatgttgagc -3’). Rescue was achieved by transducing *Capns1* KO AC2M2 cells with retrovirus produced in HEK23T cells transfected with pCL-Eco and pMSCV-Puro- *Capns1.* Calpain-1/2 protein expression and activity were assessed via immunoblotting and casein zymography, as previously described (Harper et al., 2025).

*Spheroid Invasion*

One x 10 ^4^ GFP+ AC2M2 cells were seeded into each well of a round-bottom cell-repellent 96-well plate (Greiner Bio-One, Catalogue #: 650970). After 72 hours, spheroids were collected using a p1000 pipette with sterile cut tips and resuspended in 25% Matrigel (Corning; Catalogue #: 354230) in complete DMEM. Spheroids were plated on Ibidi chamber slides (Ibidi; Catalogue #: 80426) and incubated at 37 ^o^ C for 30 minutes to allow the new layer of Matrigel to solidify. An initial image (time=0) of each spheroid was captured using an EVOS m7000 microscope on the GFP channel. Spheroids were subsequently imaged at 24, 48 and 72 hours. Quantification of invasion was performed in FIJI (Schindelin et al., 2012). The average distance invaded was estimated by calculating the mean of 4 measurements from the edge of each spheroid to the furthest point on the invasive front at 0 ^o^ , 90 ^o^ , 180 ^o^ , and 270 ^o^ positions. The total area invaded was calculated by subtracting the area of the spheroid (π x spheroid radius ^2^ ) from the area contained within the borders of the invasive front [π x (spheroid radius + average distance invaded) ^2^ ]. Within each biological replicate, data were normalized to the WT control. Statistical analyses were performed for each time point on biological replicate means using ordinary one-way ANOVA with Tukey’s multiple-comparisons test in Prism GraphPad 10.

*Resect-and-wait spontaneous metastasis*

Resect-and-wait experiments for spontaneous lung metastasis were performed as previously described (Harper et al., 2025; Hoskin et al., 2022). All experiments involving the use of animals were performed in accordance with the Canadian Council on Animal Care (CCAC) guidelines and with the approval of Queen’s University Animal Care Committee.

GFP+ AC2M2 cells were prepared in a 1:1 ratio of cell suspension (in PBS) to Matrigel. Using a 50µL Hamilton syringe (Hamilton; Catalogue #: 80500), 7500 AC2M2 cells were injected into the 4 ^th^ mammary fat pad of 8-12 week old female *BalbC-Rag2 ^-/-^ /IL2Rgc ^-/-^* mice (Colucci et al., 1999). Tumour growth was monitored beginning on day 10 using circle-template-based estimates (Staedtler; Catalogue #: 977501) and tumours were resected by recovery surgery after reaching equivalent sizes (500mm ^3^ , ~18-22 days post engraftment). Mice were euthanised 10 days post-tumour resection and lungs were harvested for imaging on the GFP Ex/Em channel on the EVOS m7000 microscope (ThermoFisher Scientific, Catalogue #: AMF7000). Anterior and posterior images of the lungs were captured, and the metastatic burden was calculated by determining the % lung area occupied by GFP+ signal. For each experimental replicate, metastatic burden was normalised to WT control. Data was analysed using ordinary one-way ANOVA with Tukey’s multiple comparisons test in Prism GraphPad 10.
